# Participatory design application in obesity prevention targeting young adults and adolescents: a mixed-methods systematic scoping review protocol

**DOI:** 10.1186/s13643-022-01900-z

**Published:** 2022-03-22

**Authors:** Taylor Jade Willmott, Alieena Mathew, Eve Luck, Sharyn Rundle-Thiele, Julia Carins, Lisa Vincze, Lauren Williams, Lauren Ball

**Affiliations:** 1 Adelaide Business School, The University of Adelaide, 10 Pulteney Street, Adelaide, South Australia 5005 Australia; 2 Social Marketing @ Griffith, Griffith Business School, Griffith University, 170 Kessels Road, Nathan, QLD 4111 Australia; 3 Nutrition and Dietetics, School of Health Sciences and Social Work, Griffith University, 1 Parklands Dr, Southport, QLD 4215 Australia; 4 Menzies Health Institute Queensland, School of Health Sciences and Social Work, Griffith University, Southport, Australia

**Keywords:** Overweight, Obesity, Obesity prevention, Participatory design, Person centred, Systematic review, User centred, Weight gain prevention, Weight management, Young adults

## Abstract

**Background:**

Prevention of obesity is economically and sociologically preferable to treatment, with early intervention key to preventing excess weight gain and obesity. The transition from adolescence to young adulthood is a critical intervention period. An expert-led, top-down model has dominated obesity prevention research and practice with limited success. Participatory design (PD) offers potential in transforming obesity prevention research and practice by delivering bottom-up solutions that young people value and may therefore voluntarily engage with over time. An evidence synthesis of PD application in obesity prevention targeting adolescents and young adults is currently lacking.

**Objectives:**

Report the protocol for a mixed-methods systematic scoping review which aims to integrate and synthesise available evidence on PD application in obesity prevention targeting adolescents and young adults. Specifically, the review will address three research questions:

RQ1: How is PD defined in obesity prevention interventions targeting adolescents and young adults?

RQ2: To what extent is PD applied in obesity preventions interventions targeting adolescents and young adults?

RQ3a: How is the utility of PD evaluated in obesity preventions interventions targeting adolescents and young adults?

RQ3b: What is the utility of PD application in obesity prevention interventions targeting adolescents and young adults?

**Methods:**

This mixed-methods systematic scoping review protocol adheres to the PRISMA-P guidelines and is informed by the PRISMA extension for scoping reviews (PRISMA-ScR). The search strategy and eligibility criteria are informed by the sample, phenomenon of interest, design, evaluation, and research type tool. Eligible studies will be peer-reviewed literature published in English, reporting on PD application in obesity prevention interventions (including intervention development, implementation, and/or evaluation) targeting adolescents and young adults (aged 10–35 years). Study designs will include qualitative, quantitative, and mixed methods. The review will comprise a systematic literature search, eligibility screening, data extraction, quality assessment using the Mixed-Methods Appraisal Tool (MMAT), and data analysis using an iterative narrative evidence synthesis approach. Evidence on PD application will be thematically integrated in terms of who was involved, when they were involved, and how and why they were involved. Further thematic analyses will be conducted according to the MATE taxonomy and the United Kingdom Medical Research Council (UK MRC’s) key functions of process evaluations. The MATE taxonomy classifies PD application in terms of methodology, agent of change, training, and engagement. The MRC describes three functions of process evaluations: implementation, mechanisms of impact, and context. Applying both in the evidence synthesis is intended to provide a more complete picture of PD application. Exploratory analyses will be conducted to assess any potential associations between PD application and effectiveness across key outcomes (weight, physical activity, sedentary time, nutrition and dietary habit, mental health, and sleep) reported within intervention evaluations.

**Conclusions:**

Elucidating PD application is a prerequisite to establishing its utility. Through the location and synthesis of available evidence on PD application in obesity prevention targeting adolescents and young adults, this review will categorise and describe different methods of PD application and explore the utility of PD application including whether any differences may be observed between PD method applied and the effectiveness of obesity prevention interventions. Implications will be delineated from the narrative evidence synthesis to inform future research and advance practice in this context.

**Systematic review registration:**

PROSPERO CRD42021268240

**Supplementary Information:**

The online version contains supplementary material available at 10.1186/s13643-022-01900-z.

## Background

The global burden of disease associated with overweight and obesity is significant, widespread, and increasing exponentially. More than 1.9 billion adults are overweight or obese worldwide [1]. The prevalence of overweight and obesity has nearly tripled since 1975 when rates first began to rise [1]. This trend shows no sign of slowing down, with a 2016 noncommunicable disease (NCD) risk factor collaboration study warning that if post-2000 trends continue, the probability of meeting the global obesity target—a halt in the rise of global obesity prevalence to match 2010 rates—is virtually zero [2]. In the absence of a successful and sustained reduction in rates of overweight and obesity, health and economic costs will reach uncontrollable levels [3]. Overweight and obesity are associated with an increased risk of developing certain chronic diseases such as cardiovascular disease, diabetes, chronic kidney disease, osteoarthritis, and some cancers [4–6].

Medical treatments, including pharmaceuticals, are not sociologically appropriate or sufficient to reduce the rising burden of overweight and obesity [7, 8], and the continuation of this approach will fail to achieve desired outcomes [9]. Reversal of rising rates of overweight and obesity around the world has yet to occur [8, 9]; thus, prevention is critical to halting future incidence of overweight and obesity [7, 10]. Australia spends AU $89 per person each year on health prevention, representing 1.34% of all health spending [11]. The lack of political will to invest in prevention is surprising given that in 2006, the prevalence cost per year for each adult with obesity was estimated at $554, and the value of an obesity cure was estimated to be $6903 per person with obesity [12]. A 2019 OECD reports find that for each US $1 invested in tackling overweight, up to US $5.6 will be returned in economic benefits [13]. Early intervention to support weight management and prevent excessive weight gain is urgently needed to deliver sustained progress over the long term [6].

Effective weight gain prevention approaches have the potential to simultaneously improve the health and well-being of young people and reduce the economic burden for society via a reduction healthcare and productivity costs associated with overweight and obesity [6, 14]. Efforts to prevent obesity have mainly focused on children, whereas other important age groups have been overlooked [15]. The transition from adolescence to young adulthood is a critical period for weight gain prevention [16]. Furthermore, the most rapid weight gain in the life course has been observed during adolescence progressing through to the early twenties to mid-thirties [15]. Incident obesity at a younger age is associated with an increased risk of chronic disease and mortality in later adult life [5, 15, 17].

Weight gain during adolescence and young adulthood has been associated with declines in physical activity, increases in sedentary behaviour, and poor dietary habits [5, 6, 18]. Higher levels of screen time and lower levels of physical activity among adolescents have been associated with lower life satisfaction and higher psychosomatic complaints [19]. For young adults, weight gain is linked to major life transitions that occur during this transitional period including moving out of home, starting work, and/or tertiary study [20]. Preventive interventions targeting behaviour change must form the primary mechanisms to reduce the growing burden of disease associated with overweight and obesity [21]. Focusing prevention efforts on the transition from adolescence to young adulthood where excess weight gain occurs is likely to yield the highest return on investment [13]. Targeted weight management approaches for adolescents and young adults have contributed to improved outcomes for this high-risk population; however, suboptimal engagement and variability in response present challenges [22]. Taken together, evidence-based programs which focus  on the unique needs and wants of the target population (adolescents and young adults) are urgently needed to halt the rising rates of overweight and obesity and improve physical and mental well-being of future generations.

Evidence reviews indicate that an expert-led, top-down model has dominated obesity prevention research and practice [23], with limited success [9]. Given that adolescence and young adulthood are developmentally unique life stages characterised by hormonal changes and cognitive development [5, 24], as well as rapidly shifting life circumstances related to home, work, family, and social relationships [20], weight management interventions targeting this transitional life stage must address both protective and risk factors. For example, prevention interventions targeting adolescents and young adults must address the factors known to contribute to excess weight gain [5, 6, 20], work to remove barriers preventing the adoption and sustained practice of healthful behaviours including environmental impediments [24, 25], and ensure adolescents’ and young adults’ needs and preferences are met within intervention design and delivery to promote continued engagement over time.

Evidence describing how best to engage and retain adolescents and young adults in weight gain prevention interventions is scant [22, 26–29]. Recent research has underscored the importance of involving intended end users (i.e. those affected by the phenomena under investigation with real lived experience) in the design, implementation, and evaluation of obesity prevention interventions [30]. End-user involvement utilising participatory design (PD) principles and methods has been suggested as an approach that may be applied to improve engagement and retention in obesity prevention interventions [30, 31]. PD is an umbrella term that encompasses a broad range of human-centred (see also citizen, consumer, person, user-centred) approaches and methods [32, 33], all varying in their extent of participant involvement [32]. PD broadly refers to the involvement of designers and users (participants) in a cooperative design process [32]. PD may represent user involvement where the audience targeted for change serve as informants (i.e. users are asked for input and feedback), or they serve as co-creator/co-designers (i.e. users are engaged as equal partners) from the outset of intervention development. Owing to its involvement of users, PD engages those affected by a given problem at the grassroots level [34]. By ensuring user needs and preferences are met within intervention design, PD offers potential for improving subsequent intervention outcomes such as user adoption, engagement, satisfaction, and retention [35].

While there is a growing evidence base indicating the involvement of those who are affected by a problem in its solution generation process can enhance outcomes achieved [36, 37], there is currently no available synthesis of evidence reporting on the utility or effectiveness of applying PD within intervention design, implementation, and evaluation of obesity prevention interventions targeting adolescents and young adults. PD offers a promising avenue to advance obesity prevention research and practice, ensuring programmes account for the knowledge, skills, and lived experience of the target audience throughout the intervention design process [34] and, more importantly, during the implementation process empowering more people to take agency in their own health and well-being [33] through the delivery of interventions more closely aligned to their needs and wants.

### Rationale

Previous reviews of obesity prevention interventions (in-person and technology-supported) targeting adolescents and young adults have focused primarily on pooling primary outcome results to obtain one overall estimate of effectiveness or have examined general trends in weight-related behaviours [38, 39]. More recent reviews have examined intervention components [40], behaviour change techniques [41], theory use [42], and external validity [28]. Limitations such as a lack of theory use [42], poor methodological quality [29, 40], lack of variation in settings outside school [29], and a general lack of external validity [28, 29] have been identified. Previous reviews of obesity prevention interventions targeting adolescents and young adults have not yet examined PD application. That is, the extent of user involvement (i.e. those affected by the phenomenon being studied) in the design, implementation, and/or evaluation of obesity prevention interventions targeting adolescents and young adults has not been systematically assessed. Consequently, the traditional expert-led, top-down model continues to dominate obesity prevention research and practice which may be limiting intervention outcomes. Application of PD offers potential in transforming research and practice in this context; however, there is a need to synthesise available evidence to establish whether a user-led, bottom-up approach elicits better results than the traditional expert-led, top-down approach.

### Research aim and questions

This mixed-methods systematic scoping review aims to locate, integrate, and synthesise available evidence on PD application in obesity prevention targeting adolescents and young adults. The review will address the following research questions:

- RQ1: How is PD defined in obesity prevention interventions targeting adolescents and young adults?
- RQ2: To what extent is PD applied in obesity preventions interventions targeting adolescents and young adults?
- RQ3a: How is the utility of PD evaluated in obesity preventions interventions targeting adolescents and young adults?
- RQ3b: What is the utility of PD application in obesity prevention interventions targeting adolescents and young adults?

## Methods

This mixed-methods systematic scoping review protocol adheres to the Preferred Reporting Items for Systematic Review and Meta-Analysis Protocols (PRISMA-P) guidelines (Supplementary File 1) [43, 44] and is informed by the PRISMA extension for scoping reviews (PRISMA-ScR) [45]. Scoping reviews are useful for identifying and mapping available evidence, clarifying key concepts and definitions, elucidating key characteristics or factors, and revealing gaps in knowledge [46]. The review will comprise a systematic literature search, eligibility screening, data extraction, quality assessment, and data analysis using an iterative narrative evidence synthesis approach. The protocol has been registered with the PROSPERO database of systematic reviews [47].

### Eligibility criteria

Eligible studies will include peer-reviewed literature published in English, reporting PD application in obesity prevention interventions (including intervention development, implementation, and/or evaluation) targeting adolescents and young adults (aged 10–35 years). Qualitative, quantitative, and mixed methods research designs will be included. No restriction will be placed on the geographical location of studies or date of publication.

For the purposes of the present review, PD will refer to any approach that engages users (participants) in the design, implementation, and/or evaluation of an obesity prevention intervention [32]. To be included, studies must clearly report application of PD or a closely related approach. Partnership-based programs not involving users will be excluded. Obesity prevention is defined as the prevention of weight gain via the maintenance of a healthy body weight and/or the reversal of small gains to maintain a healthy body weight [48]. Studies will only be included if they are explicitly focused on obesity prevention via behavioural modification. Studies evaluating weight loss or weight loss maintenance interventions with mean participant BMI > 30 kg/m^2^ will be excluded.

Adolescence is defined as individuals in the 10–19 years age group [49], and young adulthood is defined as the 18–34 years age group [50]. Thus, an age range of 10–35 years will be applied to the inclusion criteria. Studies where the population age overlaps with this criterion will be included if most (% of total sample) participants fall within the 10–35 age range. Studies that do not report an age range, the mean age of the sample, or the percentage of the sample within a given age range will be excluded. Studies involving clinical populations with existing health complications, core morbidities, or are pregnant will be excluded.

### Search strategy and information sources

The systematic search strategy is structured according to the SPIDER (sample, phenomenon of interest, design, evaluation, research type) tool [51]; see Supplementary File 2. Search terms are combined using Boolean operators. A sample search strategy for MEDLINE (Ovid) is provided in Supplementary File 2. A combination of databases is used to reliably retrieve available evidence [52]: EBSCO, Ovid, ProQuest, Scopus, Web of Science, EMBASE, and Cochrane Library (see Supplementary File 2). Databases will be systematically searched from inception (1990 onwards). The reference lists of all included papers will be searched to identify additional studies for inclusion (backward search), and Google Scholar will be used to screen papers citing included studies (forward search) for potential inclusion. Previously published systematic reviews [41, 42, 53] will also be hand searched for potentially eligible studies and check search sensitivity.

### Data management and selection process

A search log will be maintained to record the databases, keywords used, and results of each systematic database search. All records retrieved from the systematic search will be downloaded to EndNote Version X8, duplicates will be removed, and the remaining studies will be assessed for eligibility by two independent reviewers. The results will be categorised by title and abstract into the following: (i) papers appearing to meet study selection criteria, (ii) papers that should be retrieved for full-text examination, and (iii) excluded papers. The full text of potentially relevant papers will then be obtained and assessed for inclusion by two independent reviewers. At all stages, any discrepancies will be discussed and resolved via consensus with a third independent reviewer. A PRISMA flow chart [54, 55] will be produced to articulate the study selection process.

### Data collection process

A PRISMA-informed data extraction Excel spreadsheet will be used for abstracting study characteristics. Data will include study details (author, year of publication, and country), research type (qualitative, quantitative, or mixed methods), sample (sample size, characteristics, setting, retention, and blinding), data collection methods (including PD application), evaluation details (including PD measures and outcomes), and key findings and conclusions. Summary tables will be independently reviewed for accuracy and relevance in line with the MATE taxonomy of PD application [56].

### Quality appraisal/risk of bias assessment

The quality of included studies will be assessed using the Mixed-Methods Appraisal Tool (MMAT) [57, 58]. Although a variety of critical appraisal tools exist including for randomised controlled trials, non-randomised studies, and qualitative research, MMAT allows for the concurrent evaluation of methodological quality across mixed-methods research designs including mixed, qualitative, and quantitative research designs. MMAT was developed to address the challenges of critical appraisal in mixed methods systematic reviews and has been shown to be a comprehensive, useful, and reliable appraisal tool [59]. For each included study, the methodological quality can be described using the corresponding MMAT criteria, and where appropriate, an overall quality score can be calculated. Two independent reviewers will assess the quality of included studies using MMAT, with a third independent reviewer to be used in case of any discrepancies.

### Data analysis and synthesis

Data will be inductively analysed by two independent reviewers using an iterative narrative evidence synthesis approach permitting a more nuanced and fine-grained examination of included studies. In accordance with the stated research questions, the focus of the initial round of inductive analysis will be (1) PD definitions, (2) extent of PD application, and (3) utility of PD in terms of process and/or outcome evaluation. The second round of inductive analysis will focus on the extent of PD application. Extent of PD application will be integrated and synthesised in terms of who was involved, when they were involved, and how and why they were involved. A similar approach has been used previously [60]. Further thematic analyses will be conducted according to the MATE taxonomy [56] and the United Kingdom Medical Research Council (UK MRC’s) key functions of process evaluations [61]. The MATE taxonomy classifies PD application in terms of methodology, agent of change, training, and engagement. The MRC describes three functions of process evaluations: implementation, mechanisms of impact, and context. Applying both frameworks in the evidence synthesis is intended to provide a more complete picture of PD application. Exploratory analyses will be conducted to assess any potential associations between PD application and effectiveness across key outcomes (weight, physical activity, sedentary time, nutrition and dietary habit, mental health, and sleep) reported within intervention evaluations. 

## Discussion

Despite substantial investments in health and medical research, insufficient progress has been made toward halting rising rates of overweight and obesity. Combatting the growing burden of overweight and obesity will require innovative methodologies that lead to the wide-scale engagement of people who are most at risk of excess weight gain. To improve engagement and retention within obesity prevention interventions, researchers and practitioners should consider the utility in complementing the traditional expert-led, top-down model of obesity prevention with more collaborative, creative, and collective approaches that actively involve participants in intervention design, implementation, and/or evaluation. PD approaches, such as co-design, have the potential to deliver bottom-up solutions that people value and will voluntarily engage with over time. Moreover, PD provides scope for the involvement of both users and stakeholders in intervention design, implementation, and/or evaluation, thereby ensuring diverse voices are heard and reflected in solutions. While many approaches fall under the umbrella term of PD, all are underpinned by a core philosophy of inclusivity, recognising the value of engaging intended beneficiaries, users, and stakeholders in the process to arrive at a solution (i.e. an intervention) [33]. PD application has the potential to enhance intervention effectiveness by creating greater congruence between evidence-based practice and user need fulfilment within interventions. Consideration of user needs and preferences through PD is purported to optimise intervention outcomes, including user adoption, engagement, satisfaction, and retention [35]. Thus, the benefits of user involvement may extend beyond intervention effectiveness to include empowering people (allowing individuals and groups to be actively involved), democratising intervention design (imparting control over processes and outcomes to users), and promoting greater diversity (and equality) in intervention design [32, 34–36].

This mixed-methods systematic scoping review will locate and synthesise available evidence on PD application in obesity prevention targeting adolescents and young adults. Specifically, the review will classify and describe PD application, identify the extent of PD application reported within peer-reviewed literature, establish whether user involvement is assessed in process and/or outcomes evaluations, and explore whether the utility of PD application is associated with intervention effectiveness. In short, this review will provide valuable insight into which PD approaches are most often used; how they are implemented and incorporated into intervention design, implementation, and/or evaluation; and how the utility of PD is being measured or not. Moreover, an appraisal of study quality will be evaluated. Review findings will be used to formulate PD guidance for future obesity prevention efforts targeting adolescents and young adults and provide recommendations based on best practice.

## Supplementary Information

**Additional file 1.** PRISMA-P Checklist (2015): Recommended Items to Address in a Systematic Review Protocol.

**Additional file 2.** Systematic Search Strategy and Example Results.

## Data Availability

Data will be published with the review.

## Abbreviations

MATE: Methodology, agent of change, training, and engagement
PD: Participatory design
PRISMA-P: Preferred Reporting Items for Systematic Review and Meta-Analysis Protocols
PRISMA-ScR: PRISMA Extension for Scoping Reviews
SPIDER: Sample, phenomenon of interest, design, evaluation, research type
UK MRC: United Kingdom Medical Research Council
