# Supplementary material for: Participatory design application in obesity prevention targeting young adults and adolescents: a mixed-methods systematic scoping review protocol
Source: Syst Rev. 2022 Mar 22;11:51. doi: 10.1186/s13643-022-01900-z (PMC8939071; doi:10.1186/s13643-022-01900-z)
Supplement: Supplementary file 2 — Additional file 2. Systematic Search Strategy and Example Results. [file 13643_2022_1900_MOESM2_ESM.pdf]

Supplementary File 2. Systematic Search Strategy

Search Strategy

Cat.	Search terms	SPIDER
1	qualitative or quantitative or “mixed method*” or “randomised controlled trial” or “randomized controlled trial” or rct or “experiment* trial*” or “outcome evaluation” or “process evaluation” or “formative evaluation” or “community trial” or intervention* campaign* or program*	Research Type<br>Design<br>Evaluation
AND		
2	“participatory design” or “action research” or “co creat*” or “co design*” or “co develop*” or “co innovate*” or “human cent* design” or “design thinking” or “iterat* design” or “person cent* design” or “patient* cent*” or “user cent* design” or “collaborat* design” or “cooperat* design” or “user engag*” or “particp* engag*” or “patient* engag*” or “community driven” or “community led” or “community based” or “empower* evaluation”	Phenomenon of Interest
AND		
3	overweight or obesity or obese or “weight management” or “weight control” or “weight maint*” or “weight gain prevention” or “weight loss” “weight loss maint*”	Phenomenon of Interest
AND		
4	diet* or nutrit* or fruit* or vegetable* or “physical activit*” or exercise or “lifestyle” or “behaviour change*” or “behavior change*”	Phenomenon of Interest
N/A	“young adult” or “college stud*” or “university stud*” or “emerging adult*” or “young people*”	Sample (population of interest) to be manually screened following systematic searches.

Limits: English, peer-reviewed, published after 1990.
PROSPERO registration: CRD42021268240

Databases

Main Databases	Key Databases Covered
EBSCO (All databases)	CINAHL, MEDLINE
ProQuest	ERIC
Ovid (All databases)	MEDLINE, PsycINFO, PubMed
Scopus	Science Direct
Web of Science (Core Collection)	MEDLINE
Cochrane Library	
EMBASE	

Sample Search Strategy for Ovid (MEDLINE and PyscINFO)

#	Searches	Results	Type
1	((qualitative or quantitative or mixed method* or randomised controlled trial or randomized controlled trial or rct or experiment* trial* or outcome evaluation or process evaluation or formative evaluation or community trial or intervention* campaign* or program*) and (participatory design or action research or co creat* or co design* or co develop* or co innovate* or human cent* design or design thinking or iterat* design or person cent* design or patient* cent* or user cent* design or collaborat* design or cooperat* design or user engag* or particp* engag* or patient* engag* or community driven or community led or community based or empower* evaluation) and (overweight or obesity or obese or weight management or weight control or weight maint* or weight gain prevention or weight loss or weight loss maint*) and (diet* or nutrit* or fruit* or vegetable* or physical activit* or exercise or lifestyle or behaviour change* or behavior change*)).ab.	1308	Advanced
2	((qualitative or quantitative or mixed method* or randomised controlled trial or randomized controlled trial or rct or experiment* trial* or outcome evaluation or process evaluation or formative evaluation or community trial or intervention* campaign* or program*) and (participatory design or action research or co creat* or co design* or co develop* or co innovate* or human cent* design or design thinking or iterat* design or person cent* design or patient* cent* or user cent* design or collaborat* design or cooperat* design or user engag* or particp* engag* or patient* engag* or community driven or community led or community based or empower* evaluation) and (overweight or obesity or obese or weight management or weight control or weight maint* or weight gain prevention or weight loss or weight loss maint*) and (diet* or nutrit* or fruit* or vegetable* or physical activit* or exercise or lifestyle or behaviour change* or behavior change*)).kw.	0	Advanced
3	((qualitative or quantitative or mixed method* or randomised controlled trial or randomized controlled trial or rct or experiment* trial* or outcome evaluation or process evaluation or formative evaluation or community trial or intervention* campaign* or program*) and (participatory design or action research or co creat* or co design* or co develop* or co innovate* or human cent* design or design thinking or iterat* design or person cent* design or patient* cent* or user cent* design or collaborat* design or cooperat* design or user engag* or particp* engag* or patient* engag* or community driven or community led or community based or empower* evaluation) and (overweight or obesity or obese or weight management or weight control or weight maint* or weight gain prevention or weight loss or weight loss maint*) and (diet* or nutrit* or fruit* or vegetable* or physical activit* or exercise or lifestyle or behaviour change* or behavior change*)).ti.	29	Advanced
4	((qualitative or quantitative or mixed method* or randomised controlled trial or randomized controlled trial or rct or experiment* trial* or outcome evaluation or process evaluation or formative evaluation or community trial or intervention* campaign* or program*) and (participatory design or action research or co creat* or co design* or co develop* or co innovate* or human cent* design or design thinking or iterat* design or person cent* design or patient* cent* or user cent* design or collaborat* design or cooperat* design or user engag* or particp* engag* or patient* engag* or community driven or community led or community based or empower* evaluation) and (overweight or obesity or obese or weight management or weight control or weight maint* or weight gain prevention or weight loss or weight loss maint*) and (diet* or nutrit* or fruit* or vegetable* or physical activit* or exercise or lifestyle or behaviour change* or behavior change*)).hw.	63	Advanced
